# Analysis of Experimental Results Regarding the Selection of Spring Elements in the Front Suspension of a Four-Axle Truck

**DOI:** 10.3390/ma15041539

**Published:** 2022-02-18

**Authors:** Mariusz Stańco, Marcin Kowalczyk

**Affiliations:** Department of Machine and Vehicle Design and Research, Faculty of Mechanical Engineering, Wroclaw University of Science and Technology, 50-370 Wrocław, Poland; marcin.kowalczyk@pwr.edu.pl

**Keywords:** parabolic leaf spring, experimental results of front suspension, four-axle truck

## Abstract

Most special vehicles on public roads and off-road are equipped with various suspension systems. The suspensions used in trucks are designed to absorb the energy that results from overcoming uneven ground. These suspensions are divided into dependent and independent ones. Knowledge of the loads that occur while driving a vehicle, mainly off-road, is critical from the point of view of the adhesion and fatigue life of the suspension system. In the case of four-axle cars with 2 + 2 axles, in which the first two axles are equipped with a dependent suspension based on leaf springs, only one axle may carry the load. This paper attempts to analyze the results of experimental tests carried out on a vehicle in the conditions of roads with an unstable surface such as dirt roads, gravel roads, and roadless tracks. An analysis of fatigue life estimation is presented using equivalent stress values. It was also determined how the use of front axles load equalizing elements in the tested car influences their fatigue life.

## 1. Introduction

Front suspensions used in trucks operating in varied terrain must have satisfactory characteristics to achieve the required fatigue life. Leaf springs are most commonly used as suspension components on vehicles that are driven on roads with various surface conditions. They are characterized by low weight, low price, and, above all, the ability to store the energy generated by wheel movement, as required by operating conditions. Leaf spring suspensions must be characterized by adequate stiffness, strength properties of the material, and sufficient elastic deformation energy to determine their durability, which is critical in the process of a given vehicle’s exploitation, especially in challenging road conditions, e.g., construction sites, dirt roads, or unpaved roads.

In the case of two- or three-axle cars, the load on the front axle leaf spring is mainly due to the vehicle dead weight, the overload resulting from the driving speed, and the height of the bumps [1,2,3,4]. On the other hand, in four-axle cars, in which the first two axles are based on springs, there is an additional load resulting from overcoming uneven terrain [5]. This suspension is particularly susceptible to operating under fatigue-limited conditions. The suspension system is usually unbalanced statically in these cars, and there is no load equalization between the first and second axles. Exceptions are the suspensions of some Mercedes, Oshkosh, and other car models.

When driving over a high obstacle or crossing a transverse ditch by the vehicle 8 × 8, the load from the front axles (axle 1 or axle 2) is transferred to the second front axle, causing the leaf springs and vehicle frame to be overloaded. This results from the difference in the height of the wheels between the front axles (axle 1 and axle 2), which causes a differential load on the front axle (axle 1 or axle 2) leaf springs. Thus, a fatigue cycle is induced, which is key to the life expectancy of leaf springs. One of the most significant fatigue cycles, with a substantial degree of fatigue depletion in its assessment, also occurs when traversing a corrugated surface.

The data determined based on actual load runs allow one to estimate the fatigue life of suspension components. From the data obtained, it is possible to specify the load levels and character of the suspension work by indicating the load cases deciding on the durability of the suspension elements. Here, we can distinguish cases of symmetric and asymmetric suspension load. In the symmetric case, the leaf springs of the same axle are equally loaded, and the load system is symmetric with respect to the plane of geometrical symmetry of the suspension. When all wheels are in contact with the ground, the load on the axle is determined by the difference in average wheel height between the axles—the symmetrical load case. The difference in the height of the wheels on the same axle results in an asymmetric load on the suspension. In the case of asymmetric loads, the stabilizers limit the difference in the deflection of the leaf spring of the same axle. In the symmetric load case, the stabilizers do not carry any load, and therefore, their stiffness does not play a role. The asymmetric load occurs when the forces acting on the suspension elements are symmetrically distributed, with the same values but opposite signs as the effect of torsion of the stabilizers. The stiffness ratio of the leaf springs and stabilizers is then essential, and the stabilizers relieve the load on the leaf springs. 

The literature does not provide any guidelines for leaf spring loading in four-axle vehicles running on tracks with a non-uniform surface. Only Savaidis [6] refers to the works of Grubisic and Fischer [7], Grubisic [8], Rupp and Grubisic [9], Savaidis et al. [10], Lange et al. [11], and Decker and Savaidis [12], who carried out test investigations on test tracks and European public roads. He gives the following groups of loads on the front suspension components of trucks, which have a decisive impact on their strength:

- Vertical load resulting from straight-ahead driving:
  (1) $$F_{Vmax}=a*F_{n}$$

- Load occurring during braking:

(2) $$F_{Vmax}=b*F_{n}$$

(3) $$F_{hmax}=c*F_{n}$$

where:

*a*, *b*, *c*—load factors depending on the type of road (for public roads in western Europe): *a* = 2.5; *b* = 2; *c* = 1,6;
*F_n_*—vehicle axle load in relation to half of its payload.

The remaining works only concern laboratory work that involves the search for better materials [13,14,15,16,17] through numerical and experimental strength and fatigue analyses of leaf springs [18,19,20,21] or individual feathers [8,11]. Savaidis, mentioned above, also contributed to research on the fatigue life of stabilizers [22].

## 2. Suspension Description

**Figure 1:**
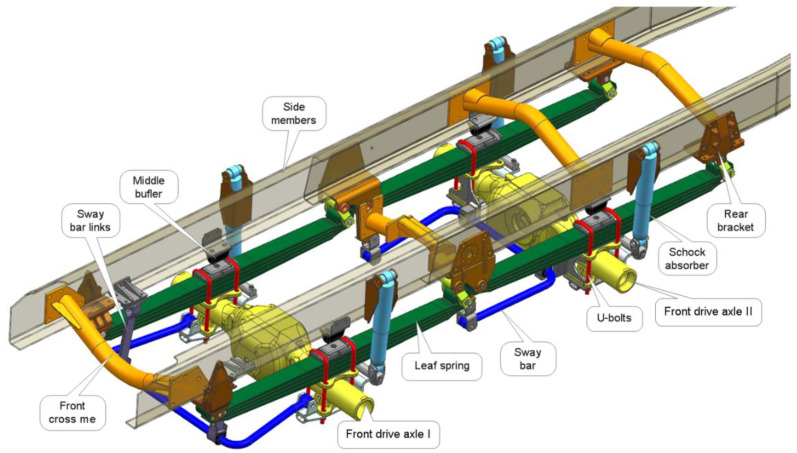
View of the suspension of the vehicle under test.

The 8 × 8 four-axle vehicle selected for the study is based on a spring suspension. The front two drive axles (Figure 1 are equipped with parabolic springs consisting of five 1600 mm long leaf springs. The rear suspension is the so-called tandem. There is no balance of forces between the first and second axles in this vehicle. When the vehicle operates on rough terrain, the spring should absorb much of the energy from overcoming an obstacle. However, the working space is limited because of the geometric limitations of the suspension and the vehicle’s subframe. The work of springs at the deflection limits is assisted by rubber buffers located in the spring center or on the chassis frame just above the axle of the bridge. The stiffness of the rubber buffers is non-linear, which means that with higher spring travel, they absorb more energy from the spring deflection, causing additional local bending in the chassis stringers. 

**Figure 2:**
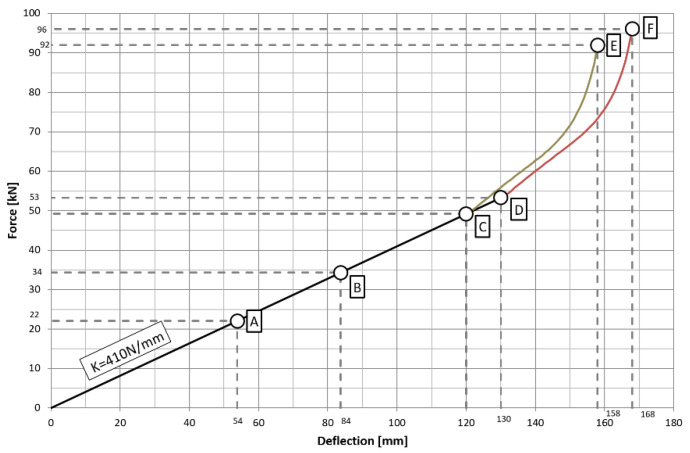
Characteristics of spring stiffness with buffers.

In the case of the vehicle in question, the leaf springs have a stiffness of Kr = 410 N/mm. In the event of asymmetric loading of the leaf springs, they are supported by two stabilizers of different stiffness. The first axle stabilizer has a torsional stiffness of K1 = 126.8 kN/mm, while the torsional stiffness of the second axle stabilizer is K2 = 100.7 kN/mm. The characteristic operating points of the suspension are shown in Figure 2. Point A is the static spring load of axle I. Point B is the static load on the axle spring of axle II. Points C and D are the leaf spring-to-buffer contact moment. Points E and F represent the maximum deflection of the leaf spring.

Figure 1 shows the structure of the suspension system and frame in isometric view.

**Figure 3:**
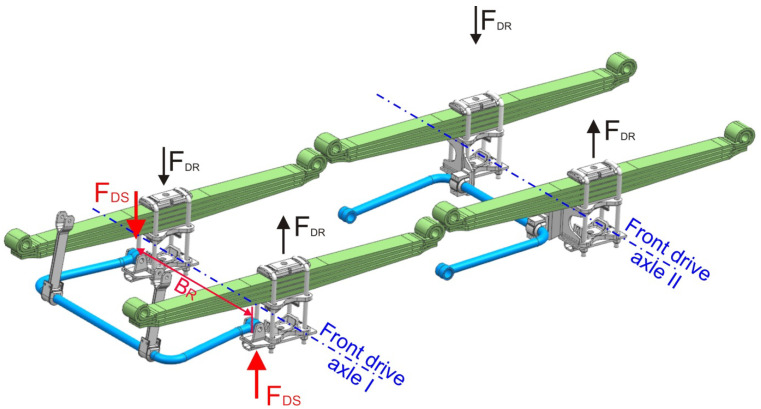
Defined forces of the asymmetric load case in the first and second axle suspension systems.

There are two similar stabilizer systems with different dimensions in the vehicle chassis. In the case of axle one, the spacing of the stabilizer mounting points to the bridge is identical to that of the BR springs (Figure 3). The displacements of the leaf springs relative to the frame are almost identical to the displacements of the stabilizer mounting points. The differences in the displacement points of attachment of the stabilizer to the frame result, among other things, from the angle of twist of the frame, which depends on the displacements of all four axes. Displacements of the suspension mounting points of the other three axles affect the displacement differences points of attachment of the stabilizer to the frame. They are one of the factors that cause fluctuations in the asymmetric load pattern of the first front axle leaf springs (signal *C*1–*C*2 in relation to the stabilizer load pattern (signal 2 × *C*6). The second factor that influences the variation of the courses of the spring load differences (*C*1–*C*2) in relation to the stabilizer load (2 × *C*6) is the geometric nonlinearity of the stabilizer mechanism.

In the case of the second axis, the distance between the stabilizer fixing points and the bridge is different than B_R_ (Figure 3), but the defined F_DS_ forces have a spacing of B_R_. This change in the system of forces was taken into account in the value of the stiffness K_2_.

Two cases of axle suspension loading were defined:

- LCS—the case of symmetrical spring loading, in which the stabilizer does not generate forces and the elastic forces of the *F_R_* and *F_L_* suspension elements in the leaf spring spacing coincide with their average value:

(4) $$F_{M}=\frac{F_{R}+F_{L}}{2}$$

- LCD—the case of asymmetric spring loading, in which the stabilizer generates a pair of forces, and the elastic forces of the suspension elements produce the difference in *F_D_*:

(5) $$F_{D}=\frac{F_{R}-F_{L}}{2}$$

The force in the suspension elements in the leaf spring spacing on the right side is:

(6) $$F_{R}=F_{M}+F_{D}=F_{M}+F_{DR}+F_{DS}$$

and on the left-hand side:

(7) $$F_{L}=F_{M}-F_{D}=F_{M}-F_{DR}-F_{DS}$$

where:

*F_DS_*—stabilizer force acting in the leaf spring spacing;
*F_DR_*—differential leaf spring force.

In signal analysis, the sum *C*1 + *C*2 corresponds to the force F_M_, and the difference in *C*1 − *C*2 corresponds to the sum of *F_DR_* and *F_DS_* (on axis I). To assess the importance of the stabilizer, it is helpful to compare the distributions of the ranges of variation of the individual *C*1 and *C*2 signals with their mean value. The comparison would correspond to comparing the distributions of the ranges of variation of the quantities measured on the axle springs with the range of variation of half the axle load.

## 3. Measurement System

**Figure 4:**
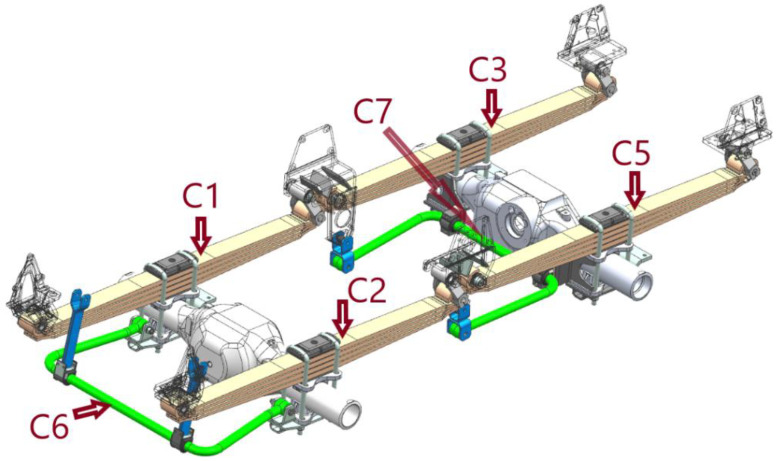
Measurement points on the elements of the suspension system of the first and second axles.

A measurement system was developed and constructed for load testing. It was adapted to long-term measurements under polygon conditions. The synchronous measurement of eight physical quantities was foreseen on the test object. Strain gauges were selected to measure the loads on the springs (channels *C*1, *C*2, *C*3, and *C*5). They were located at the same distance from the spring axle for each leaf. Since the vehicle was driven in tough terrain on unpaved roads, all sensors were placed on the upper surface of the central leaf on the stretched side. Deformations of the stabilizer rods of the first and second axle (*C*6 and *C*7 channels) were also measured. Additionally, vertical accelerations were calculated (*C*4 and *C*8 channels). The measuring sensors were placed on the vehicle frame just above the first axle. The selected measurement points and their location are shown in Figure 4 (without *C*4 and *C*8 channels because points were located on the truck frame).

For strain measurements, inverted HBR strain gauge half-bridges with a strain gauge constant *k_SG_* = 2.19 were used. In each of these measurement paths:

- A pair of strain gauges on one rosette was glued to the structural member and temperature-compensated;
- The additions to the strain gauge half-bridges were located just outside the transducer inputs and were made from strain gauge pairs glued on steel plates to provide temperature compensation;
- The use of half-bridges inverted from the negative pole of the power supply made it possible to lead the differential measurement signal with a twisted pair of wires and protect the recorder against cases of mechanical damage of the wire and the measurement point;
- The average strain in two active strain gauges was measured;
- The reference voltage of the A/C converter was equal to the supply voltage of the bridge of the strain gauges;
- The gain *G* was 128.

**Figure 5:**
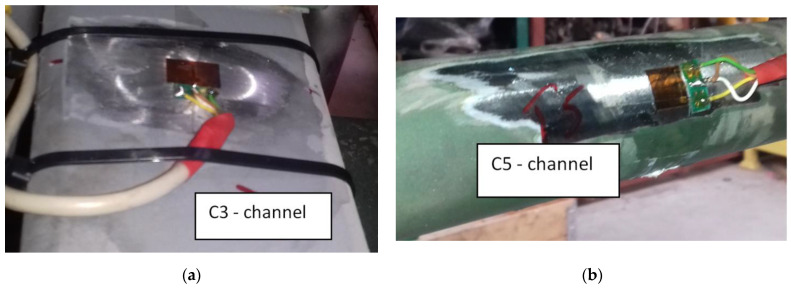
View of developed test points (**a**) on the leaf spring; (**b**) on the stabilizer bar.

The measuring points were installed on an unloaded car. This was carried out to determine the loads caused by the vehicle’s operation; therefore, it was not necessary to install them in an unloaded state. A view of the developed measuring points is shown in Figure 5.

In the case of the *C*1, *C*2, *C*3, and *C*5 measuring tracks, the first direction of strain measurement at the measuring point was in accordance with the direction of stress $\sigma_{B}$ due to bending of the spring blade. The second direction of strain measurement at the measuring point was perpendicular to the first direction. The strain measurement fields were in the middle of the spring leaf width, and the effect of spring leaf torsion was compensated within the pair of active strain gauges. The post-conversion value $w_{B}$ in the measurement paths of the springs can be calculated from the relation.

(8) $$w_{B}=k_{SG}\cdot \frac{\sigma_{B}}{E}\cdot \frac{1+\nu }{4}\cdot G\cdot 2^{R-1}$$

where:

E—longitudinal modulus of elasticity of steel;
ν—Poisson’s number;
R—bit resolution of the A/C converter equal to 16.

In the *C*6 and *C*7 measurement paths, the average deformations caused by the effect of torsion of the cylindrical part of the stabilizer were measured. Strain gauges were positioned along the directions of linear strain measurements caused by tangential stresses from torsion $\tau_{S}$. The value after conversion in the measurement paths of stabilizers $w_{S}$ can be calculated from the relation:

(9) $$w_{S}=k_{SG}\cdot \frac{\tau_{S}}{E}\cdot \frac{1+\nu }{2}\cdot G\cdot 2^{R-1}$$

In the case of torsion, the measuring path sensitivity is twice as high as in the case of bending.

## 4. Initial Results Evaluation

The measurement data collected on the computer’s SSD drive were periodically downloaded via the Internet connection. The technical condition of the measurement system was also assessed. Before the final processing, the measurement data were subjected to preliminary analysis. The analysis did not reveal any irregularities in the signal waveforms of the strain gauge measuring paths. The signals of the measuring tracks were grouped into two similar groups assigned to the axes:

- The first: *C*1 and *C*2 of the pair of leaf springs, *C*6 of the stabilizer between the springs;
- The second: *C*3 and *C*5 pairs of leaf springs, *C*7 stabilizer between springs.

Within each group, it was found that:

- The sum of spring signals is proportional to the average axle load and spring deflection;
- The difference of spring signals is proportional to the difference in spring loads and spring deflection;
- The difference in spring signals is approximately proportional to the stabilizer signal;
- After one-off subtraction of the signal offsets, no signal shifts caused by temperature drift were found.

The zero level of signals was assumed for the vehicle at standstill on a flat horizontal ground loaded with its own weight.

**Figure 6:**
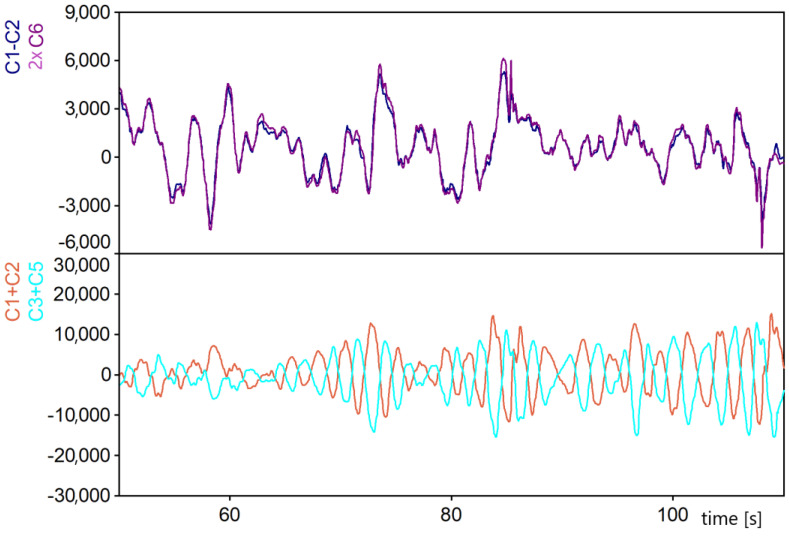
Value waveforms after conversion of selected measurement tracks.

The preliminary analysis of the recorded waveforms used the values after conversion. They were then processed according to relation 13 and 14. The waveforms were compared to the example waveform (Figure 6): the waveforms of the difference of values after conversion in the measuring paths *C*1 and *C*2 on the springs with the doubled value after the conversion of *C*6 on the stabilizer between the pair of springs of the first axle almost coincided. The small discrepancies in the signals may be due to the not fully accurate kinematic relationship between the displacement of the springs and the angle of twist of the stabilizer in the suspension mechanism. An analogous situation occurred in the case of the second axle. The waveforms of the difference of values after the conversion of *C*3–*C*5 almost coincided with the waveform of the doubled value after the conversion of *C*7.

The waveforms of the sum of values after conversion in measuring paths of the first axle *C*1 + *C*2 and the second axle *C*3 + *C*5 were compared. The waveforms shown in the example in Figure 6 are as if they are mirror images, which indicates that the axle load levels alternated during the drive, but the differences in spring deflections between the left and right sides were almost completely cancelled out in the sum values. The mean values of the highlighted signal sums are twice as low.

The preliminary analysis of the waveforms allowed us to assess the data quality and conclude that:

- The measuring system is well made and functions stably;
- There are proportions between the measured quantities in the strain gauges, which are reflected in the waveforms;
- The relations between the values of the measured quantities are the same within both groups of gauges, and there is no need to make corrections in the scaling of the gauges; the scaling constants for the gauges on the springs are identical and so are the stabilizers;
- In the case of a defect in one of the measurement paths within the group, it is possible to reconstruct the signal from the remaining two signals.

The example of the waveforms in Figure 6 shows the nature of the suspension loads on the two front axles of the vehicle. The load variation of the springs within an axle is completely independent of the mean value of the axle load. In general, the observed variation ranges of spring load differences are several times smaller than the variation ranges of their mean values.

## 5. Measurement Results

**Figure 7:**
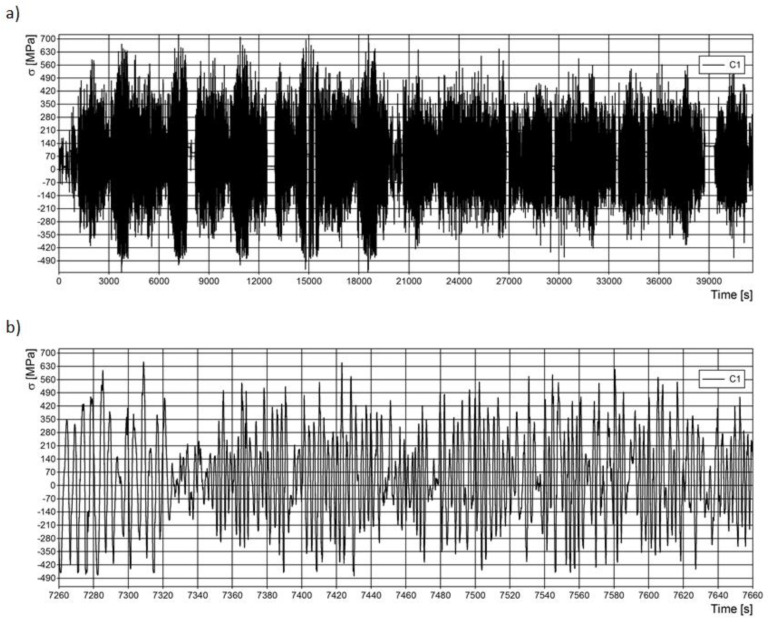
Stress curves in point *C*1: (**a**) whole curve; (**b**) enlarged curve—400 s of measurement.

The measurements were taken over the distance of approximately 10,000 km in the autumn. During the tests, the vehicle was loaded to its maximum total mass (32,000 kg). It was driven on asphalted and dirt roads with a hard surface and on unpaved roads with an often deteriorated surface and bumpy ground. The average daily mileage of the vehicle on asphalt roads was about 500 km, while on dirt roads, it was about 120 km. During the tests, the vehicle was driven at a speed that allowed safe driving in off-road conditions. The maximum speed was about 25 km/h. The measurement data received were used to obtain strain waveforms, from which the stress waveforms in the measurement directions were determined. The measurement results obtained in the test section during one measurement day are presented below. Example stress curves measured at point *C*1 are shown in Figure 7. 

A statistical analysis was performed using the obtained results. It consisted in determining the distribution of stress ranges for each measurement point. The stresses in the cases of symmetric and asymmetric loading of the springs were calculated from the relationship:

(10) $$\sigma_{M1}=\frac{\sigma_{C1}+\sigma_{C2}}{2}\;\mathrm{and}\;\sigma_{M2}=\frac{\sigma_{C3}+\sigma_{C5}}{2}$$

(11) $$\sigma_{D1}=\frac{\sigma_{C1}-\sigma_{C2}}{2}\;\mathrm{and}\;\sigma_{D2}=\frac{\sigma_{C3}-\sigma_{C5}}{2}$$

where:

$\sigma_{M1},\;\sigma_{M2}$—symmetrical load of axle I and axle II;
$\sigma_{D1},\;\sigma_{D2}$—asymmetrical load of axle I and axle II;
$\sigma_{C1},\;\sigma_{C2},\;\sigma_{C3},\;\sigma_{C5}$—load recorded at points *C*1, *C*2, *C*3, and *C*5.

**Figure 8:**
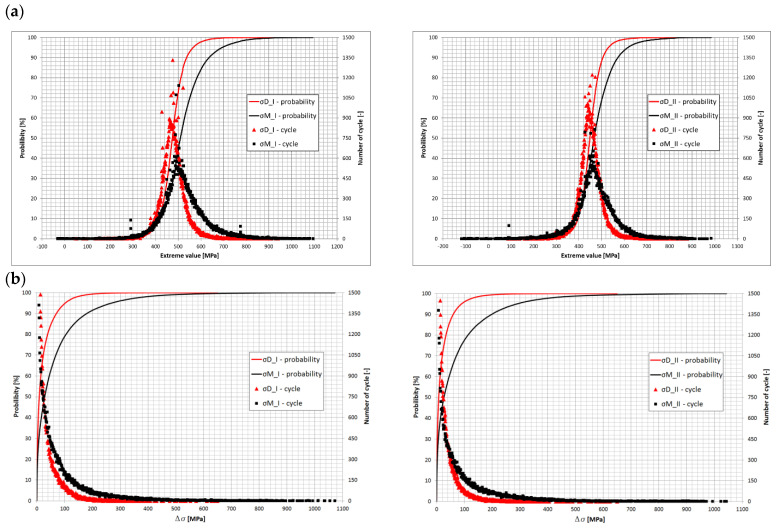
Symmetric and asymmetric stress distributions of both axle springs and the corresponding numbers of cycles of (**a**) alternating stress (**b**) stress ranges.

Based on the obtained stress distribution curves of the leaf springs, it was found that the leaf springs carried the load in the range Dσ ≈ −200 – 200 MPa. The static load of leaf springs was about 450 MPa. The value is significant for further consideration of the fatigue life of leaf springs. Loads above 200 MPa or below −200 MPa occurred occasionally (Figure 8a). 

The determination of symmetrical stresses $\sigma_{M1}$ and $\sigma_{M2}$ provides information on the average stress of axles I and II springs. On the other hand, unsymmetrical stresses *σ_D_*_1_ and $\sigma_{D2}$ provide information on the stress difference between the left and right sides of the car. The data thus obtained give information on the operation of the stabilizer. From such data, it is possible to determine whether the springs worked evenly during the car’s life and what effect the stabilizer will have on the life of the spring. Figure 8a shows the developed distributions of the symmetric and asymmetric stress waveforms together with the number of occurrences of extreme stress values and the number of occurrences of the stress ranges Dσ (Figure 8b).

**Figure 9:**
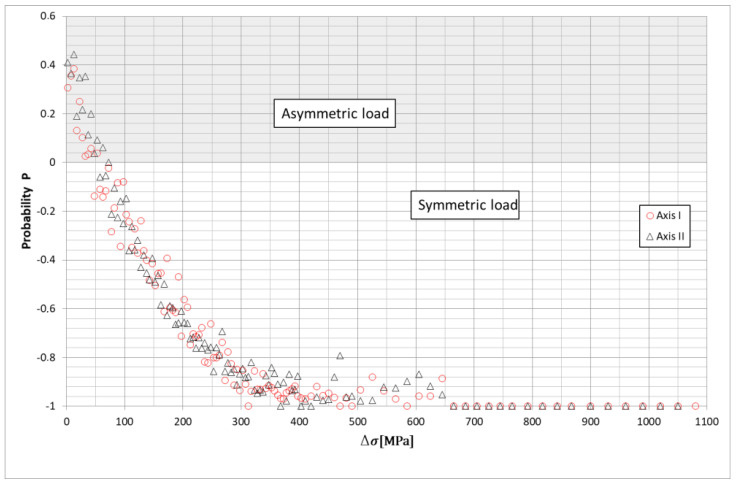
Comparison of probabilities of the occurrence of cases with corresponding stress ranges for both axes for symmetric and asymmetric examples.

**Figure 10:**
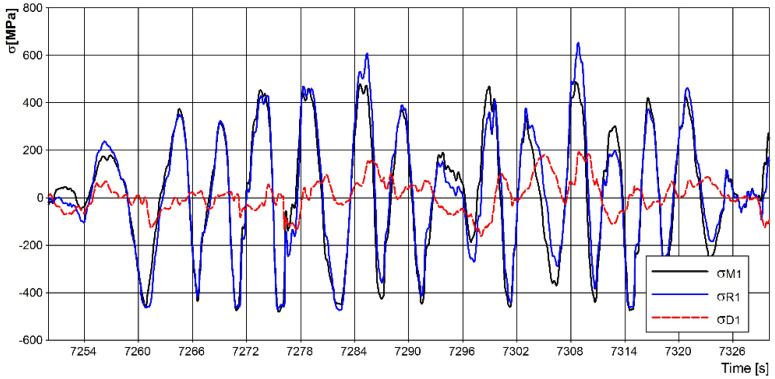
Stress waveform for point *C*1 together with symmetric stresses *σ*_*M*1_ and asymmetric stresses $\sigma_{D1}$.

The presented distributions with stress ranges show that stress values with amplitudes above 100 MPa were much less frequent for asymmetric loading cases, indicating that the spring was simultaneously bent under symmetric (higher proportion) and asymmetric (lower proportion) loading during asymmetric (lower proportion) loading operation. From the graphs presented, their nonuniform operation can be observed. For the entire test section, the predominant criterion affecting the magnitude of stresses in individual spring pairs was uniform spring bending, as shown in Figure 9. For the asymmetrical loading of both springs, there were more cycles for ranges up to about 30 MPa. Above this value, significantly more cases occurred for symmetrical bending of the springs. The courses of symmetrical stresses, asymmetrical stresses for the first axis, and the stresses recorded at point *C*1 are shown in Figure 10. From the presented course, it is possible to notice a slight difference in the courses of symmetrical stresses and the stresses recorded at point *C*1, marked on the diagram as *σ_R_*_1_.

## 6. Load Estimation for Fatigue Life Determination

The obtained results were subjected to fatigue life analysis to determine (check) the life of the spring by analyzing the results for each measuring point and the results of the whole axle. The Rainflow method was used to count the cycles. The theoretical fatigue life was then estimated using the Palmgren–Miner linear damage accumulation method.

**Figure 11:**
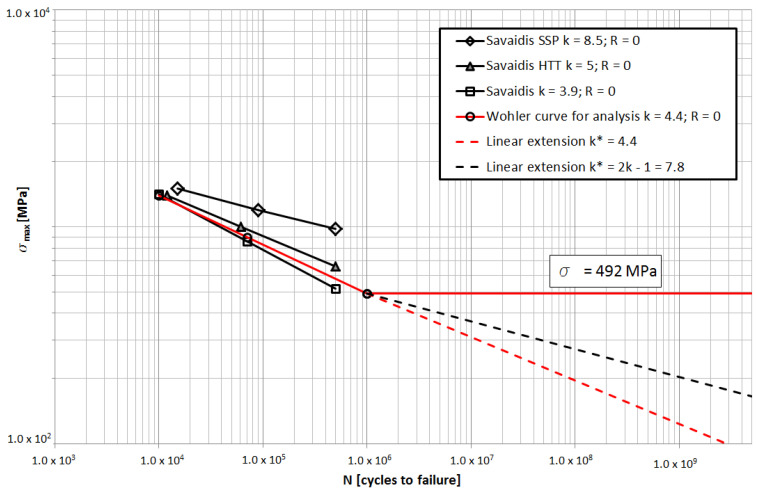
Fatigue curves of 51 CrV4 steel for R = 0 and the characteristic (red) adopted based on the performed tests (red).

The material from which the springs were made is 51 CrV4 steel. Data to develop a fatigue curve were selected based on papers [1,21,23] and the results of tests carried out on leaf springs. Figure 11 shows the fatigue curves for 51 CrV4 steel with different values of the exponent *k* depending on the state of the material after the spring manufacturing process at *R* = 0. The material characteristics adopted for the analysis of the measurement results are presented in the diagram. To sum up the damage, the characteristic curve was extended while maintaining the k-value below the high-cycle fatigue limit. Since the literature lacks information about the behavior of this type of steel under loads with low stress amplitudes, it was assumed (based on the article [24]) that the fatigue life of the spring steel for the number of cycles *N* > 10^6^ continued to decrease linearly to the value ¼ of the high-cycle fatigue limit.

**Figure 12:**
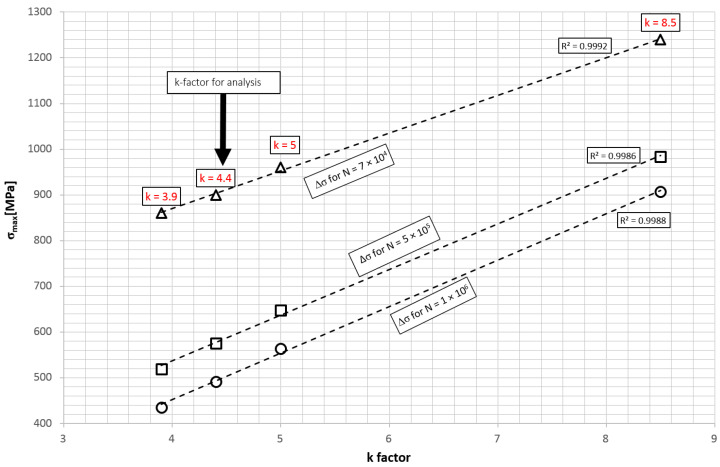
Change of maximum stress σ_max_ at constant number of cycles for various *k* values.

The relation between different values of the k-factor (Equation (12)) for the same cycle asymmetry factor R was analyzed. Data for the same steel grade were taken into account. Figure 12 shows the way the value of the maximum stress *σ*_max_ changes for different factors *k* in a similar number of cycles *N*. By running a straight line through the points obtained, a linear relationship between the stress range and the *k* factor for different numbers of cycles can be observed. The fatigue limit, defined as the knee point on the Wohler curve was taken to *N_k_* = 1 × 10^6^ cycles [9]. Below the knee point, the *k*-factor was checked for k* = *k* and for k* = 2*k* − 1 [25].

(12) $$k=\frac{logN_{2}-logN_{1}}{log\sigma_{a1}-log\sigma_{a2}}$$

**Figure 13:**
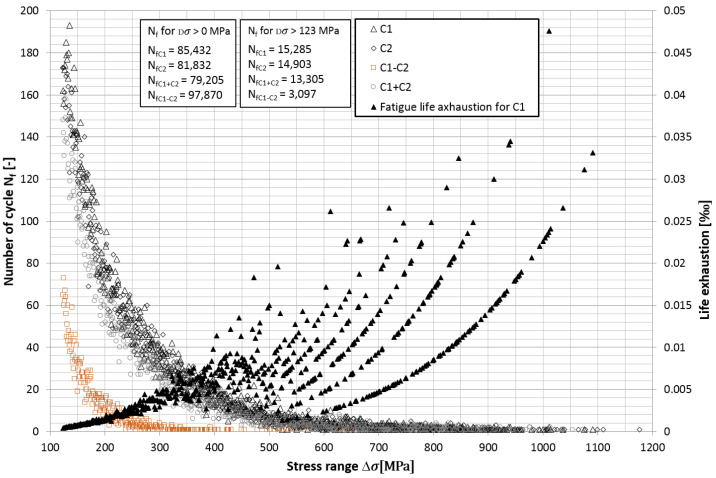
Number of cycles and fatigue life exhaustion of leaf springs of axis I.

Given the assumed fatigue characteristics, using the aforementioned linear method for determining the accumulation of damage, an analysis of the exhaustion of spring life was carried out. Figure 13 shows the number of cycles for both axle I spring and the number of bending cycles in the cases of symmetrical and asymmetrical suspension loads. The proportion of the number of cycles with small amplitudes for stresses in the range of unlimited fatigue strength (below 123 MPa) is very high and is about 83%. For axis I symmetrical cases, it is 77%, while for asymmetrical cases, it is as high as 93%. The durability of the tested vehicle’s springs is limited by the stress cycles in the range above 300 MPa, and the use of the modified characteristics in the breakdown of damage does not prejudge the comparison of the fatigue degree.

In the diagram, as mentioned above, the exhaustion of fatigue life for the measuring point *C*1 is shown on the right vertical axis. For amplitudes of stresses above Dσ > 400 MPa, the fatigue life limits are arranged in several ascending curves instead of one. This is the consequence of the vehicle overcoming different amplitude bumps.

To assess the influence of various factors on the durability of the leaf springs, the authors proposed the use of equivalent stress values. In the analysis of the results, it is necessary to fulfil the following criteria:

1. Cycle number values can only be compared for the same stress range Dσ.
2. The values of stress ranges Dσ can only be compared for the same number of fatigue cycles.
3. It is possible to convert the stress ranges or the number of fatigue cycles in a way that does not change the exhaustion of the fatigue life for a given fatigue cycle. The results are converted according to the characteristics of the spring material (Figure 11).

In the case of fatigue cycle with the number of cycles *N_a_* and stress range ∆*σ_a_*, the parameters of this fatigue cycle can be converted to equivalent *N_b_* and ∆*σb* from relation (13):

(13) $${\left(\frac{\Delta \sigma_{a}}{\Delta \sigma_{b}}\right)}^{k}=\frac{N_{b}}{N_{a}}$$

Then, based on the analysis of the results and the obtained distributions, equivalent stress ranges were calculated according to Miner’s linear Palmgren damage accumulation (14):

(14) $$\Delta \sigma_{e}^{k}=\sum_{i=1}^{n}\frac{\Delta \sigma^{k}*N_{i}}{N_{e}}$$

where:

Dσ*_e_*—equivalent stress range (MPa);
Dσ—stress range (MPa);
*k*—Wöhler curve factor (-);
*N_i_*—number of cycles for a given stress range Dσ (-);
*N_e_*—total number of cycles ∑*Ni* (-);
*n*—number of intervals of stress range.

After transformation, Equation (14) takes the form:

(15) $$\Delta \sigma_{e}=\sqrt[{k}]{\sum_{i=1}^{n}\frac{\Delta \sigma^{k}*N_{i}}{N_{e}}}$$

Equivalent stress ranges and number of cycles were determined by assuming that the k-factor is constant, which may be debatable. The aim of the calculations is not to precisely indicate the fatigue or service life of the leaf springs but to assess the stage of fatigue life through loading individual components of the leaf springs—symmetrical and asymmetrical parts and the influence of the suspension system design. In the assessment, the ratios of fatigue life depletion are significant. In the case of the research object, the highest contribution to fatigue life depletion is stress ranges > 300 MPa.

In each case of the determination of equivalent stress ranges, the value of the stress ranges for different numbers of cycles was obtained. To enable the comparison of the number of cycles, they were recalculated according to relation (13), which, after transformation, can be written as follows:

(16) $$N_{e_{2}}={\left(\frac{\Delta \sigma_{e}}{\Delta \sigma_{e_{2}}}\right)}^{k}\cdot N_{e}$$

where:

$N_{e_{2}}$—number of cycles for $\Delta \sigma_{e_{2}}$[-];
$\Delta \sigma_{e_{2}}$—equivalent stress range for number of cycles $N_{e_{2}}$[MPa];
$\Delta \sigma_{e}$—equivalent stress range determined from measured data [MPa];
$N_{e}$—estimated fatigue life based on linear damage accumulation for equivalent stress $\Delta \sigma_{e}$ [-].

The calculation of the equivalent value is correct when it relates to the points remaining in the fatigue characteristic (Figure 11); for example, where one value of the exponent k applies. 

**Figure 14:**
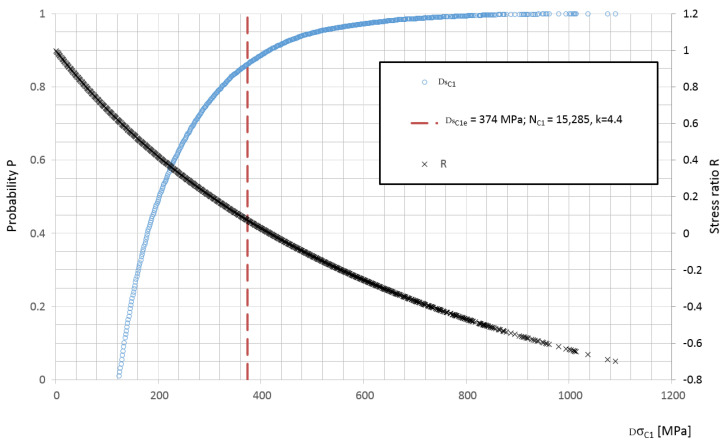
Distribution function of stress ranges P, equivalent value for point *C*1 and distribution of cycle asymmetry factor R.

The equivalent value of the stress ranges is always assigned to the number of cycles for specific stress value level. Using the Wöhler curve factor k, the equivalent value can be recalculated for the other cycle numbers. Cycle numbers can also be calculated by changing the level of the equivalent value of a stress range so that comparisons of cycle numbers are possible. An example of the location of the equivalent value for point *C*1 is shown in Figure 14. The change of the R value for the given stress ranges was also specified. To determine this value, it was assumed that the mean value of the stress in the leaf spring is constant, as the measurements were carried out on a vehicle with constant mass throughout the tests. Eventually, the truck was expected to run with a constant load.

The ratio of the number of cycles of the symmetrical component of the run to the number of cycles of the full run of a given spring is evaluated. The sequence in achieving this is as follows:

1. Determination of the equivalent number of cycles and the equivalent stress range of the full fatigue run for one spring according to Equation (14).
2. Determination of equivalent number of cycles and stress range for the symmetrical part of the run in the same way as in item 1.
3. Recalculation of equivalent number of cycles for the same stress range according to Equation (16).
4. Calculation of the ratio of the equivalent number of cycles of the symmetric component to the number of cycles of a full course from one spring.

The percentage of life depletion due to the existence of asymmetric component of the waveform can be written on the example of the right axle one spring:

(17) $$DN_{1R}=\left(1-\frac{N_{1M}}{N_{1R}}\right)*100\%=\left(1-\frac{D_{1M}}{D_{1R}}\right)*100\%$$

where:

$N_{1M}$—number of cycles of symmetrical part of the first axis waveform;
$N_{1R}$—number of runs registered on the first axle spring on the right side of the vehicle;
$D_{1M}$—degree of fatigue life depletion in the symmetrical part on axle I;
$D_{1R}$—degree of fatigue life depletion in the right leaf spring of axle I.

The remaining DN shares were calculated analogously for the other springs. On the *N*_1*M*_ position of the equation, there is the value of the number of cycles of the symmetrical part of the run, and on the *N*_1*R*_ position, there is the number of cycles of the full run for a given spring.

The DN values (Table 1) indicate the key role of the symmetrical part of the load path for the front axle springs. The leaf springs are identified by the test track signals (Figure 4) or by the way these signals are converted for further consideration. In a system like the one in the test vehicle, where there is no spring load equalization system, the DN contributions of the asymmetric part of the waveform to the life depletion are on average 11% for the first axle (*C*1 and *C*2) and 18% (*C*3 and *C*5) for the second axle. The fatigue of the springs is determined by the symmetrical part of the spring load course. There are visible DN differences between springs of the same axle caused by running asymmetrically on dirt roads. 

An attempt was made to assess what difference a spring load equalization system on the same vehicle could make to the situation. Based on the same measurement results, after processing the measurement signals, equivalent stress ranges and cycle numbers were calculated as before for the spring elements of the suspension when using load-equalizing elements on the individual wheels. Such elements are used in trailers, tractor-trailers, tandem suspensions, and some multi-axle trucks.

In a suspension system with a spring load equalization system, the stress level in the two springs on the right side of the vehicle was calculated from the relations:

(18) $$\sigma_{R}=\frac{\sigma_{C1}+\sigma_{C3}}{2}$$

and on the left side:

(19) $$\sigma_{L}=\frac{\sigma_{C2}+\sigma_{C5}}{2}$$

In turn, the stress value of the symmetrical component of the stress course from the relation was calculated:

(20) $$\sigma_{ME}=\frac{\sigma_{C1}+\sigma_{C2}+\sigma_{C3}+\sigma_{C5}}{4}$$

It is impossible to faithfully reproduce the trajectory of vehicle movement and unevenness in the subsequent field test. Only the analysis of the results of processed signals according to the principle of suspension operation allows a comparison of suspension systems of different designs to be made with minimal influence of changes in operating conditions. The spring load equalization system under consideration would result in equal loads on a pair of springs on the same side of the vehicle. On the right-hand side in both springs, the spring load would be equal to the mean value resulting from the average of the *C*1 and *C*3 signals. On the left-hand side in both springs, the load value would be proportional to the mean value of the *C*2 and *C*5 signals. The two mean values calculated for the right and left springs represent the complete load pattern of these springs in a suspension system equipped with spring load-balancing systems. In such a suspension system, the symmetrical part of both waveforms is common to all springs and is the average of the loads on the four springs, i.e., the average of the *C*1, *C*2, *C*3, and *C*5 signals. 

The result of such an experiment may be subject to error since the spring load equalization system causes the values of spring load extremes to get closer to each other as the wheels adhere to the ground and deform it more uniformly. On the one hand, a decrease in spring load extremes will reduce tire deflection and ground deformation, while increasing the spring’s contribution to energy absorption. On the other hand, an increase in spring load minima will have the opposite effect. Therefore, it can be concluded that if the same experiment could be replicated in field testing, the result should be similar to that in the calculations.

Table 1 shows the individual equivalent stress values for each measuring point as well as for the mean value of the symmetrical load and the elements in which the axle balancing took place. Given the above, the corresponding equivalent cycle numbers were determined. The expected number of cycles was then estimated for the reference level (200 MPa). For the individual measurement points, the influence of the asymmetrical component of the waveform on the process of life depletion was also specified. For the first axis, this will average around 11%, while for the second axis, it will be 18%. Finally, the effect of axle balancing on the fatigue life of the springs is shown. It falls in the range of 768–1700 times.

When planning the experiment, predictions were made, which were confirmed by the analysis results (Table 1).

1. There was a decrease in the number of load cycles on the springs due to the exclusion of the effect of unevenness of the ground height under the wheels on the same side of the vehicle; the number of cycles for the original system is kN times higher (Table 1) by an average of 220 times. The degree of durability increase kN depends on the exponent *k* and grow with increasing *k*. A significantly high value of kN shows how important the design of the suspension system is for a special off-road vehicle.
2. There was an increase in the proportion of asymmetrical part of the load curve for DN springs due to a decrease in the stress range; the increase was also noticeable in DN values averaged 6.2 times for the first axle springs and averaged 3.9 times for the second axle springs.

**Figure 15:**
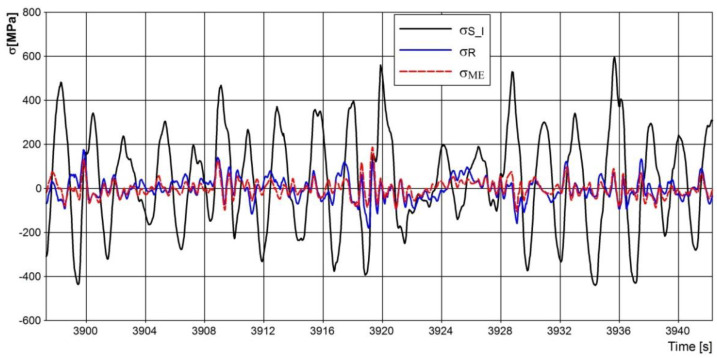
Stress distribution in leaf springs.

Figure 15 shows a fragment of the stresses in the springs with axle balancing. The use of a load equalization system for the leaf springs on both sides of the vehicle significantly reduces the maximum level of stress ranges and at the same time extends their fatigue life.

The influence of a material with different characteristics than for *k* = 4.4 (Figure 11) on a fatigue life was also verified. The DN values included in Table 2 were calculated based on the fatigue life depletion stage D in relation to the material with *k* = k* = 4.4 and for *k* = 4.4 and k* = 2*k* − 1 = 7.8. Using a material with the highest *k*-factor increases the leaf springs life by more than 20 times.

## 7. Discussion

In the correct selection of leaf spring elements for the truck suspension system, knowledge of the loads resulting from the operation of such a vehicle plays an important role. The next step is the selection of appropriate materials, which will be characterized by the desired adequate strength and the desired fatigue life. It is not obvious what combination of properties of suspension components is desired. It is not always possible to freely select dimensions of suspension geometry and design. By studying the load courses of suspension elements, it is possible to indicate the key cases in the exhaustion of durability of loads and properties of suspension elements and the appropriate directions for design development in challenging, unusual cases in which the vehicle will be operated. 

Based on the conducted research, it was noticed that the stress ranges occurring in the leaf spring elements of the front suspension system in the studied four-axle truck on roads with uneven ground are very high. However, during their analysis, the focus was not on their magnitude, but rather on the influence of the symmetrical and asymmetrical parts on the fatigue life of the components. 

In the assessment method that consists of calculating the equivalent cycle number ratios, only the k-factor is relevant. The use of the modified characteristic instead of the characteristic with constant *k* does not impact the values of the cycle number ratios. In the case of the fatigue life depletion degree D, the remaining material properties and the use of a variable *k* highly influence the results of the depletion degree calculation, but its analysis is not the subject of this paper. An enhancement of the leaf spring durability can be achieved by improving the strength properties of the material. In the case of an analyzed suspension system for a special-purpose vehicle, the highest increases in durability were obtained by adjusting the design of the suspension system. The effect of different spring mounting methods on fatigue life was analyzed. The load equalization system of the front axle leaf springs on both sides of the vehicle result in an increase in the springs’ durability by hundreds of times. The implementation of such a system is complicated by the displacements that occur at the spring mounting points. The equalization system would have to ensure that the spring mounting points move within a range twice as large as the difference in wheels displacement. However, a very high value of kN (Table 1) indicates that the spring load equalization over the full range of wheel displacement is not necessary to achieve increased durability. The degrees of fatigue life depletion increase in a system with a modified design can be estimated by recalculating the values of stresses in the leaf springs according to the relations describing any suspension mechanism.

It has been shown that asymmetric loading has a relatively small effect on leaf spring durability. Increasing the stiffness of the stabilizers and the torsional rigidity of the frame does not solve the problem of low leaf spring fatigue life limit. The cycles of the symmetrical part of the stress that range above 300 MPa are mainly responsible for the fatigue life depletion of the leaf springs in off-road vehicles. The desired service life of the leaf springs can mainly be achieved by limiting the level of the maximum stress ranges. It is not necessary to use a system that completely equalizes the load on the leaf springs on the same side of the vehicle front axle. A material with a higher *k* is more sensitive to higher fatigue cycles, so introducing the system that partially limits the maximum stress ranges gives more effective results when *k* is higher.

In the literature, there are study results of a material that (depending on manufacturing processes), achieves *k* in the range of 3.9 – 8.5. It was observed that there is an approximately linear relationship between *k* and the stress in the strength range limited to a selected number of cycles. This relationship was not confirmed by the authors’ tests. The *k* factor of the leaf spring (test object) material was determined to be 4.4, and its properties are similar to the materials with *k* value 3.9 and 5.

## 8. Conclusions

The research work carried out and the conclusions drawn from it can be summarized as follows:

- Information on the distribution of stress ranges in leaf springs during truck operation is significant in assessing the durability of the vehicle’s suspension. The results presented in this paper show that in special operating conditions where numerous cycles occur in the limited life range, they determine the fatigue life of the leaf spring.
- Through several analyses, it was proven that in the suspension system without equalizing the spring loads, the asymmetric part of the load has little effect on the shortening of the fatigue life, and thus, the stiffening of the torsion bars will not significantly improve the service life of the leaf springs. The main load affecting the service life of the spring is the symmetrical load.
- Equivalent stress ranges calculated according to the fatigue characteristic curve with constant k-factor (k* = *k*, Figure 11) enable an initial and quick assessment of factors influencing leaf spring durability, such as: suspension system design, and asymmetric load component. The exponent *k* = 5, typical for leaf spring materials, can be used in the initial assessment. Values of DN and kN indexes (Table 1 and Table 2) are underestimated when using fatigue characteristics with a constant k-factor, so it is advisable to calculate them more precisely using modified fatigue characteristics.
- The introduction of a system limiting the levels of maximum stress ranges results in a many times higher increase in leaf spring life than the improvement in material properties. A system that allows at least the partial equalization of front axle loads makes better use of material with higher fatigue properties.

## Figures and Tables

**Table 1:** Summary of the durability comparison results for material with k* = *k* = 4.4.

Measuring Path Signals		*C*1	*C*2	*C*3	*C*5	$\frac{C1\;+\;C2}{2}$	$\frac{C3\;+\;C5}{2}$	$\frac{C1\;+\;C3}{2}$	$\frac{C2\;+\;C5}{2}$	$\frac{C1\;+\;C2\;+\;C3\;+\;C4}{4}$
Equivalent stress	Dse (MPa)	374	380	414	412	378	406	167	174	160
Number of cycles for the comparative level Dse_cp Dse = 200 MPa	Ne	241,070	251,927	366,113	368,909	218,215	300,613	776	845	168
Contribution of the asymmetrical component of the load course on depletion of life	DN (%)	9.5 9.5 *	13 13 *	18 19 *	19 19 *	-	-	78 77 *	80 90 *	-
Multiplicity of life increase in the leaf spring load balancing system	kN	310.8 4528 *	298.1 2054 *	472 7285 *	436.5 3154 *	-	-	-	-	-

* calculated for k* = 2*k* − 1 (Figure 11).

**Table 2:** Estimated degrees of leaf spring durability increase when using a material with different fatigue characteristics compared to the base material (k*= 4.4 = *k*).

*k*	DN*_C_*_1_	DN*_C_*_2_	DN*_C_*_3_	DN*_C_*_5_
3.9	−0.33	−0.32	−0.28	−0.29
	−0.34 *	−0.33 *	−0.28	−0.29
**4.4**	**0.00**	**0.00**	**0.00**	**0.00**
5	0.62	0.59	0.49	0.50
	0.64 *	0.60 *	0.49 *	0.51 *
8.5	32.7	28.4	20.7	22.3
	44.6 *	36.2 *	24.2 *	26.2 *

* calculated for k* = 2*k* − 1 (Figure 11).

## Data Availability

Not applicable.
